# Prime editing – an update on the field

**DOI:** 10.1038/s41434-021-00263-9

**Published:** 2021-05-24

**Authors:** Janine Scholefield, Patrick T. Harrison

**Affiliations:** 1 Department of Human Biology, University of Cape Town, Cape Town, South Africa; 2 Bioengineering and Integrated Genomics, NextGen Health, CSIR, Pretoria, South Africa; 3 Department of Physiology, University College Cork, Cork, Ireland

**Keywords:** Genetic engineering, Targeted gene repair

At *Gene Therapy*, we are continually monitoring the landscape of research, noting those technologies which advance the goal of clinical translation of the field. In one of the most exciting ‘needle-shifting’ gene editing publications published in 2019, David Liu et al., (who pioneered base editing; BE [1]), developed ‘Prime editing (PE) [2]’, erasing several limits of CRISPR that have caused bottlenecks in its therapeutic and biotechnological applicability. Traditional gene editing strategies directing specific changes to the genome sequence itself largely reply on a common step – creating a double-strand break (DSB) to attract and exploit the DNA repair pathway machinery in executing the desired repair. This necessary ‘injury’ to the cell concomitantly contributes to many of the concerns in shifting gene editing to the clinic. This is where BE, and now PE, present technological inflection points on the road to advancing patient treatment. Put another way, say goodbye to DSBs and hello nicks. Thus, with the increased precision potential of PE, we can now accelerate clinical gene editing even further.

In this perspective, we review the basics of PE and discuss some of the advances that have been made in pushing this method and broadening its applicability since the seminal publication.

## Back to basics – the operational components of PE

**Fig. 1:**
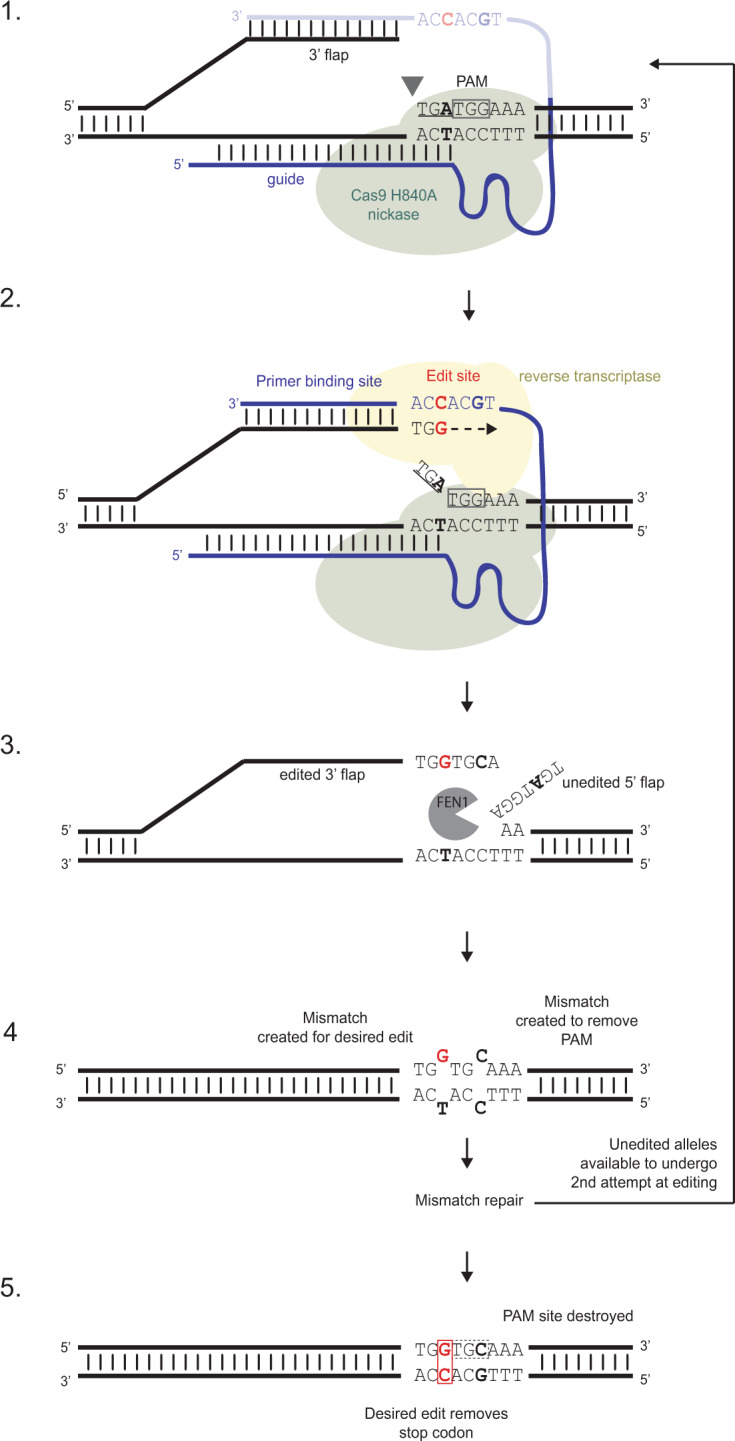
Prime editing in five steps. Prime editing has just two components, a Cas9 nickase fused to a modified reverse-transcriptase (referred to as PE2) and a multifunctional prime editing guide RNA (pegRNA). **1** The Cas9-H840A/pegRNA complex binds to the desired target region and creates a nick 3 bp upstream of the PAM site. The nick must be *upsteam* of the first variant site (in this case a TGA stop codon) and occurs on the same strand as the PAM liberating a 3’ flap. **2** This 3’ flap forms a sequence-specific interaction with the 14-16 nt “primer binding site” located at the 3’ end of the pegRNA. This RNA/DNA hybrid serves as the PRIMEr site for new DNA synthesis using the RNA “edit site” as a template; the modified RT polymerase copies the template thereby extending the 3’ flap. **3** The edited 3’ flap displaces the variant unedited 5’ flap, which is removed by a cellular nuclease FEN1. **4** In this example, this leaves two MisMatches to be resolved, one in the edited codon (G ≠ T), and one in the modified PAM (C ≠ C) which can be introduced as an option to prevent further editing to the corrected sequence. **5** MisMatch Repair resolves the DNA resulting in either precisely edited DNA (with no indels), or the original variant sequence. In the latter case where the PAM sequence has not been modified, the Cas9-H840A/pegRNA complex can bind to the variant sequence again and have another attempt at PRIME editing. **Key**: Cas9-H40A nickase shown in Green, Reverse Transcriptase in yellow. The PAM site is highlighted in a grey box; the pegRNA in blue; the 3’ edit site in red; the edited PAM site in bold; FEN1 in grey. For clarity, only part of the pegRNA is shown in step 1.

The PE system retains CRISPR’s targeting specificity but carries with it, additional cargo in the form of an edit-containing RNA template as a contiguous extension of the guide RNA (known as the pegRNA), and M-MLV reverse transcriptase (RT) fused to the C terminus of Cas9 (H840A) nickase. Use of the Cas9 nickase avoids the formation of a DSB, and simply cuts the non-complementary strand of the DNA three bases upstream of the PAM site. This exposes a DNA flap with a 3’ OH group which binds to the primer binding site (PBS) of the RNA template, serving as a primer for RT, which extends the 3’ flap by copying the edit sequence of the pegRNA (Fig. 1). Despite this extended 3’ flap being thermodynamically less likely to hybridise to the unedited complementary strand compared to the unedited 5’ flap, the inherent preference of the endogenous endonuclease FEN1 to excise 5’ flaps leads to hybridisation of the edited 3’ flap being favoured, thus resulting in highly efficient base editing. And this was PRIME version 1.0.

By engineering a series of changes into the RT sequence (PE2) significant improvements in editing efficiency were observed with very low levels of random insertions/deletions (indels). The final step in the editing process is the resolution of the short heteroduplex region of DNA that occurs as a result of direct editing of just one strand of DNA. This can resolve naturally in favour of the desired editing process with reasonably high efficiency. But the authors showed that co-transfection of a standard gRNA targeting the complementary strand allows the H840A Cas9 to nick the non-edited strand, and this biases mismatch DNA repair in favour of the edited sequence by using the edited DNA strand as a template to complete the process. The only downside to the use of this PE3 strategy is a slight increase in indel formation due to both DNA strands being nicked at roughly the same time. To solve this issue, a gRNA can be designed to recognise the complementary strand of DNA only *after* the PE3 edit has occurred. In the small number of cases tested, this revised strategy (PE3b), can increase editing but with reduced indel formation, essentially to the level of that seen with PE2 editing.

The clever combination of engineered molecular biology components results in a number of advantages, in addition to high efficiency of editing by single base substitution. Whilst their previous BE strategies provided a mechanism of creating single base substitutions for the four transitions (C > T; T > C; A > G; G > A), and recent studies have expanded this to include two transversions (C > G and G > C [3–5]), PE encompasses all potential 12 modifications including the eight transversions. This provides a much swifter process for developing therapeutic editing for any disease caused by a single base pair change, as well as the potential for researchers to model any SNP in vitro. PE further allows efficient production of small deletions and insertions broadening the scope of this tool to addressing almost 90% of disease-causing mutations. In one case, PE3 was shown to introduce single base changes up to 34 bp downstream of the nicking site, the consequence of which is that the NGG PAM can be positioned quite some distance from the target site. This further allows for a single pegRNA to correct different mutations in a hotspot region of a gene. The therapeutic implication is that the same pegRNA could be used to correct a small range of disease-causing variants in a number of different patients. By way of example, in cystic fibrosis, two of the three most common PTC variants, G542X and R553X (neither of which can be treated by currently available CFTR modulators) are only 33 nucleotides apart in the same exon, so a single pegRNA could potentially correct either variant.

The second major advantage of the PE system is that it mitigates the need for double-strand break (DSB) repair machinery, which is notoriously error prone. Liu et al. demonstrated that indels were a rare event, significantly outnumbered by the prevalence of precise edits measured as a percentage of total sequencing reads - an inversion of what is experienced with classic Cas9 homology-directed repair (HDR) where indels are often the predominant outcome [6]. (If you’re a numbers person, whilst indels still occur, the frequency is at least one log less than HDR). Furthermore, off-target effects observed at predicted regions of the genome were almost undetectable in comparison to Cas9 DSB-dependent repair systems. These results are of significant interest since undesirable changes in the genome (caused by both targeted indels and off-target effects) has been a significant concern to taking therapeutic gene editing to the clinic.

Additionally, the original study described an exciting experiment, which overcame a significant hurdle in repairing post-mitotic cells. Since almost all precise repair strategies generally require repair templates, they must exploit the endogenous HDR machinery, restricted to dividing cells. This has been a bottleneck in therapeutic applications of gene editing, especially for the many neurological diseases involving mutations that affect post-mitotic neurons. However, as PE bypasses the need for HDR machinery, precise genome substitutions were observed (albeit at low frequency) in primary mouse cortical neurons. With many diseases shown in preclinical trials to be alleviated by overcoming minimum threshold levels of cellular repair, even modest levels of gene correction may effect clinical improvement.

When this seminal paper was published, it was considered to potentially be one of the most promising developments in the field of gene editing since CRISPR was first discovered [7]. However, as researchers around the world have rushed to acquire the PE plasmids, additional data is revealing just how amenable this refined molecular tool really is to advancing therapeutic genome engineering, as well as some inherent difficulties in applying the PE system. For instance, even in its simplest form it is highly modular – thus requiring optimisation of multiple components. In addition, as has been the case with many sequence-specific technologies, significant optimisation of the pegRNA is required.

Addressing these and other issues, a number of recent studies have explored how PE can be used to efficiently repair and model disease-causing variants in cells, organoids and mice embryos, and a number of new online tools have been developed to help design pegRNAs. In the following sections, we outline some of these advances.

## Editing mammalian cells and organoids

### Efficiency of repair

One of the primary aims of any GE advance is efficiency of repair as it dictates the delivery strategy in approaching the clinic i.e. higher repair efficiency may provide clinical benefit from in vivo systemic delivery, whilst lower efficiencies may necessitate an ex vivo approach. Since PE is not reliant on HDR, the efficiency of corrected alleles is an important yardstick with which new studies can provide critical information. Schene et al. evaluated PE3 editing in human stem cells to model disease in organoid culture models and reported efficient targeted deletions close to the PAM site, and induction of a specific transversion (C > G) modification at the +26 position [8]. For two loci examined, they observed editing efficiency of 30–50%. They also showed correction of disease-causing in-frame deletions and frameshifts with concomitant restoration of normal function in cell models of Wilson’s disease and *DGAT1* deficiency with efficiencies of >20%.

A study by Sürün et al. demonstrated that PE also works efficiently (3–6%) in human iPS cells, converting eGFP to CFP by targeting a di-nucleotide sequence [9]. In a pre-print, Rousseau et al. report that the use of cytosine BE to create the Alzheimer’s disease-causing Ala673Thr variant in the APP gene was successful, but was accompanied by numerous bystander C > T modifications [10]. In contrast, PE enabled them to precisely make the Ala673Thr variant, although the efficiency of editing was much lower than CBE. Of note, they further reported that the second round of PE could be used to increase editing efficiency. Guerts et al. report on BioRxivs that PE could correct the most common CF-causing mutation, but was >30-fold less efficient than HDR using a selection-based screening assay [11]. They further performed a head-to-head comparison of Adenine BE, Prime and HDR using the Arg785X CF-causing variant, and show that ABE was ≥6-fold more efficient than either PE or HDR.

### Ratios of correct editing to unwanted byproducts

The ‘cost’ of efficient editing can often be measured by the assessment of unwanted byproducts including both bystanders and indels. The Schene et al. study critically revealed that unwanted byproducts at the pegRNA or PE3 sgRNA target sites occurred at low rate of 1–4%, in both stem cells and cell lines – an order of magnitude lower than the desired editing efficiency [8]. In addition, in a head-to-head comparison for correction, the ratio of correct editing to indel formation was shown to be 30-fold higher for PE relative to Cas9-HDR, similar to values reported by Anzalone et al. [2]. This appears to reveal a common theme. In their preprint, Kim et al. attempted to correct a G > A mutation at the 3’ end of exon 8 of the *Fah* gene which disrupts splicing [12]. Three different ABE/gRNAs combinations were tested and bystander editing of neighbouring A’s occurred at a higher level that the target A. In contrast, PE corrected the mutation with no detectable bystander effect, despite the overall efficiency being lower than editing with ABE.

### Off-target effects

A third (and perhaps the most important) factor acting as a bottleneck in translating GE technologies to the clinic lies in predicting and evaluating off-target effects. The absence of DSB formation promises to impart a significant layer of safety to PE-based therapies. Critically, in two independent experiments, Schene et al. failed to detect any off-target edits after performing whole-genome sequence analysis.

Collectively, these studies reinforce that where BE is possible without bystander effects, then BE will invariably perform better than PE - an observation originally noted by Anzalone et al. However, if the target is a frameshift, indel or site with potential bystander targets, PE is the most suitable option with much higher editing to indel ratio than HDR.

## Editing in mice embryos

The original PE paper demonstrated not only highly efficient corrections in many cell types but importantly revealed very low indel mutation rates [2]. This is particularly important in vivo, and was explored by Liu et al. who used the PE3 system in correcting a mutation in the *Hoxd13* gene in mouse one-cell embryos homologous to a clinically relevant gene in humans [6]. Analysis of edited blastocysts revealed editing in the range of 1–19% which gave rise to pups with varying levels of editing in different tissues indicating somatic mosaicism.

In a wider ranging preliminary study of PE in mice, Aida et al. use PE3 editing to target six different loci [13]. Whilst they report efficient editing, they describe a high frequency of undesired outcomes, particularly deletions corresponding to the spacing between the two nicks. Upon removing the nicking sgRNA, the PE2 approach eradicated deletions but revealed reductions in editing. Working within the same loci, but at different sites, the PE3b approach resulted in efficient editing such that the undesired indels were no longer observed in the majority of cases.

Recently Gao et al. compared HDR and PE2 to edit the CArG box transcription factor binding site in mice [14]. Analysis of founder mice revealed approximately twice as many successfully edited animals with HDR (20/37) versus PE2 (12/47). However, no spurious on-target edits were seen in PE2 animals whereas many HDR founders had variable levels of on-target indels. In addition, 5 of 11 HDR founder mice had off-target edits whereas none were reported in the PE2 animals.

## Design and optimisation of pegRNAs

The original study by Anzalone et al. highlighted the need to optimise the pegRNA, specifically recommending the testing of different pegRNAs with different PBS and RTT lengths. Several recent reports have further analysed pegRNA design, and reach similar conclusions with minor refinements. For example, from an analysis of a high throughput evaluation of PE2 editing, Kim et al. recommend the following pegRNA: a 13-nt PBS and 12-nt RTT, a GC-rich PBS and incorporating a G as the last templated nt if the RTT is ≤12-nt [15]. They also stress the importance of, where possible, using the RTT to disrupt the PAM, an approach already utilised in several of the examples above. It has to be acknowledged however that the PBS length is likely to be dependent on sequence context as some studies demonstrate PBS lengths of 10–12 to be most effective [8, 9].

Fortunately, design tools such as multicrispr, which is compatible with PE and is potentially future-proofed for new editing tools in the pipeline such as Cas9-transposases are now available [16]. Other published webtools include pegfinder, which selects sites and provides sequences of oligos for cloning [17].

Two studies describe automated designs for pegRNAs cross-referenced with the ClinVar database to both correct disease-causing variants for therapeutic use, or create them to model disease [18, 19]. In addition, Hsu et al. show PrimeDesign can be used for genome wide and saturation mutagenesis screens. PrimeDesign allows the user to design PE3b nicking guideRNAs (ngRNAs), which disrupt either the seed sequence of the ngRNA spacer, or disrupt the non-seed sequence of the ngRNA spacer. The authors suggest that PE3b seed ngRNAs may exhibit greater specificity in nicking the non-edited strand after the edited strand flap resolution and may thus be more suitable than PE3b non-seed ngRNAs.

## Engineered Cas9-RT with increased PAM flexibility

The modular nature of PE can be considered a double-edged sword in that whilst a number of components must be optimised, specific pieces can be ‘swapped out’ to ensure adaptability. To potentially increase the utility of PE2 editing, Kweon et al. substituted different Cas9 variants with altered PAM specificity and assessed their impact on PE2 editing at a range of different genomic sites [20]. They reported successful editing with variants referred to as PE2-SpG, PE2-NG and PE-SpRY, thus expanding the coverage of targetable pathogenic variants in the ClinVar database that can now be prime edited to 94.4%.

## Conclusions

Whilst the seminal paper by Anzalone et al. describing PE garnered a great deal of excitement for those of us in the gene therapy field, it was acknowledged that the multiple components necessary to execute PE required significant optimisation. What is clear from our perspective is that in just over a year a plethora of studies is advancing this tool by producing user-friendly design tools, which will enable researchers to harness this method. Encouragingly, we see data emerging that PE works in multiple cell types, organoids and mice embryos. Interestingly, there are also indications that editing in embryos may employ slight differences in DNA repair pathways. PE has also had a significant impact on the bioengineering of plants, where prime-editing efficiency appears linked to optimising pegRNA expression, but these are discussed elsewhere [19].

Despite perhaps being the second choice to BE in a few scenarios, with its ability to target >90% of pathogenic variants in the ClinVar database, it seems inevitable that PE is destined for clinical implementation in the not too distant future. As with the exciting appearance of CRISPR Cas transposases [21] advancements in PE will be continue to be monitored by *Gene Therapy* with great anticipation.
